# Catalytically Competent Non-transforming H-RAS^G12P^ Mutant Provides Insight into Molecular Switch Function and GAP-independent GTPase Activity of RAS

**DOI:** 10.1038/s41598-019-47481-1

**Published:** 2019-07-29

**Authors:** Metehan Ilter, Ozge Sensoy

**Affiliations:** 1 Istanbul Medipol University, The School of Engineering and Natural Sciences, Department of Biomedical Engineering, Istanbul, 34810 Turkey; 2 Istanbul Medipol University, The School of Engineering and Natural Sciences, Department of Computer Engineering, Istanbul, 34810 Turkey

**Keywords:** Computational models, Molecular conformation, Molecular conformation, Oncogenes, Oncogenes

## Abstract

RAS mutants have been extensively studied as they are associated with development of cancer; however, H-RAS^G12P^ mutant has remained untouched since it does not lead to transformation in the cell. To the best of our knowledge, this is the first study where structural/dynamical properties of H-RAS^G12P^ have been investigated -in comparison to H-RAS^WT^, H-RAS^G12D^, RAF-RBD-bound and GAP-bound H-RAS^WT^- using molecular dynamics simulations (total of 9 *μ*s). We observed remarkable differences in dynamics of Y32. Specifically, it is located far from the nucleotide binding pocket in the catalytically-active GAP-bound H-RAS^WT^, whereas it makes close interaction with the nucleotide in signaling-active systems (H-RAS^G12D^, KRAS4B^G12D^, RAF-RBD-bound H-RAS^WT^) and H-RAS^WT^. The accessibility of Y32 in wild type protein is achieved upon GAP binding. Interestingly; however, it is intrinsically accessible in H-RAS^G12P^. Considering the fact that incomplete opening of Y32 is associated with cancer, we propose that Y32 can be targeted by means of small therapeutics that can displace it from the nucleotide binding site, thus introducing intrinsic GTPase activity to RAS mutants, which cannot bind to GAP. Therefore, mimicking properties of H-RAS^G12P^ in RAS-centered drug discovery studies has the potential of improving success rates since it acts as a molecular switch *per se*.

## Introduction

RAS proteins are guanine nucleotide-dependent molecular switches which participate in cell proliferation, differentiation, growth, and survival^1–6^. Three human RAS oncogenes (H-RAS, K-RAS, and N-RAS) encode four proteins, namely H-RAS, N-RAS, K-RAS4A and K-RAS4B, which are comprised of 188- or 189 amino acids, having a sequence identity of 90% or more among them^7–9^. The activity of this protein family is modulated by the phosphorylation status of the nucleotide. That is to say, when RAS is bound to guanosine triphosphate (GTP), it is activated and can initiate several cellular signalling pathways such as Raf/MEK/ERK or PI3K/Akt^10–12^. Upon hydrolysis of GTP to guanosine diphosphate (GDP), which is mediated by GTPase activating proteins (GAPs), RAS is inactivated and signaling is terminated^13^.

Since RAS protein family is involved in crucial cell processes as mentioned above, mutations are closely associated with development of various cancer types such as lung, bladder, pancreas, and colon^14,15^. They are generally seen in the 12^th^, 13^th^, and 61^st^ residues^8,15^. In particular, the mutation that occurs in the 12^th^ residue is the most frequent mutation in K-RAS and H-RAS subtypes. Moreover, the G12D mutation is the most prevalent one among three frequent G12 mutations (G12C, G12D and G12V)^8^. They cause loss of intrinsic or GAP-mediated GTPase activity of the protein. Therefore, the mutant RAS is locked in the signaling-active state and cannot terminate signal. The underlying structural cause of this functional change has been revealed by elucidation of crystal structures of various G12 mutants of RAS subtypes^16–23^. On the other hand, the mechanistic insight could only be provided by means of molecular dynamics studies. Lu *et al*. have shown that G12 mutants of K-RAS4B such as G12C, G12D and G12V cause inactive-to-active conformational change by triggering different dynamics in RAS^8^. Compared to K-RAS4B^G12C^, K-RAS4B^G12D^ and K-RAS4B^G12V^ distort the nucleotide binding pocket more in the GTP-bound state, whereas K-RAS4B^G12C^ causes larger conformational change in the GDP-bound state which leads to higher exposure of the nucleotide^8^. In another molecular dynamics study, G12D and G12V oncogenic mutants of H- and K-Ras have been comparatively studied and H-RAS^G12V^ has been shown to be more flexible than its K-RAS counterpart. In the same study, it has been also shown that H-RAS^G12V^ mutant adopted a conformational state that prevented protein from interacting with the effector^24^. Moreover, Vatansever *et al*. have shown that GTP-binding stabilizes motions in K-RAS and leads to residue correlations having long decay times^25^.

In spite of close association of RAS mutants with development of cancer, H-RAS^G12P^ mutant does not lead to cancer in the cell (non-transforming mutant). Similar to transforming RAS mutants, H-RAS^G12P^ mutant also cannot bind to GAP since pyrrolidine ring of P12 points outward and prevents interaction between RAS and GAP effector^8,16,26–29^; however, it can still hydrolyze GTP using its intrinsic GTPase activity. Structurally, transforming H-RAS^G12D^ (PDB ID: 1AGP)^16^ and non-transforming H-RAS^G12P^ mutant (PDB ID: 1JAH)^17^ are very similar as the backbone RMSD between them is measured as 0.44 Å and increases up only to 1.4 Å when considering the side chain atoms as well. Consequently, this suggests that non-transforming mutant displays different dynamics that provides intrinsic GTPase activity to the protein, hence can be used as a model system to understand molecular mechanism of GAP-independent GTP hydrolysis.

In this study, we performed comparative analysis of structural and dynamical properties among H-RAS^G12D^, H-RAS^G12P^ and H-RAS^WT^ by means of long atomistic molecular dynamics simulations (total of 9 *μ*s). Trajectories of GAP-bound and RAF-RBD-bound H-RAS^WT^ were used to represent the catalytically- and signaling-active states of the protein, respectively. We showed that non-transforming H-RAS^G12P^ mutant displays remarkable differences in terms of dynamics of the system. Specifically, Switch I and II are more stable in the non-transforming mutant which leads to have a more compact nucleotide binding pocket than the transforming mutant. Considering that Switch II is responsible for interaction with nucleotide exchange factors (GEF) the stability of this region increases the strength of interaction between RAS and GEF, thus expediting the rate of nucleotide exchange as in agreement with experimental data^16^. Interestingly, Y32, which has been shown to stimulate GTPase activity of RAS^30^, displays different dynamics in signaling- and catalytically-active states. It is positioned closer to the nucleotide in the former and in wild type RAS, whereas it is located far from the nucleotide in the latter. Moreover, the accessibility of Y32 in wild type protein increased upon GAP-binding. On the other hand, Y32 samples both of these states in the GTP-bound non-transforming mutant *per se*, and the accessibility of the residue decreases in the GDP-bound state, which agrees with experimental data^30^. Considering i) the role of Y32 in intrinsic GTPase activity of RAS^31,32^, ii) the relation between incomplete opening of Y32 and the onset of cancer^33^, and iii) undraggibility of RAS^34–36^ we propose that Y32 can be used as an alternative site that can be targeted by means of small therapeutic molecules to displace it from the nucleotide binding pocket, thus introducing intrinsic GTPase activity to transforming RAS mutants, which cannot bind to GAP.

## Results

In this study, we carried out comparative analyses of local and global structural/dynamic properties of H-RAS^WT^/H-RAS^G12D^/H-RAS^G12P^ (wild-type/transforming mutant/non-transforming mutant), GAP-bound and RAF-RBD-bound H-RAS^WT^ by means of atomistic molecular dynamics simulations to provide mechanistic insight into transforming activities of these systems. For H-RAS^WT^ and H-RAS^G12P^, we studied GDP-bound states as well. RAF-RBD-bound and GAP-bound systems were used to represent signaling- and catalytically-active states of the protein, respectively.

### Comparison of root-mean-square fluctuation (RMSF) profiles reveals significant differences in residues with functional relevance

**Figure 1:**
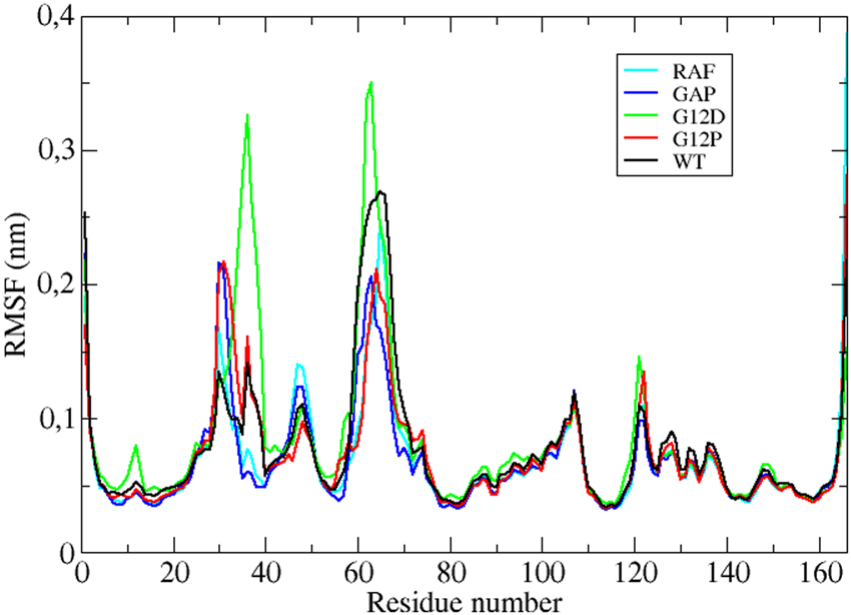
Root-mean-square fluctuations of H-RAS^G12D^ (PDB ID: 1AGP), H-RAS^G12P^ (PDB ID: 1JAH), GAP-bound H-RAS^WT^ (PDB ID: 1WQ1), RAF-RBD-bound H-RAS^WT^ (PDB ID: 4G0N) calculated from molecular dynamics trajectories of GTP-bound systems.

Comparative RMSF analysis of the systems shows remarkable differences in fluctuation patterns of mutant RAS proteins. Specifically, the mutation of glycine to proline residue increases stability more in H-RAS^G12P^ than H-RAS^G12D^. In particular, T35 and G60 which coordinate *γ*-phosphate of the nucleotide, and Q61 that participates in the catalytic activity of RAS less fluctuate in H-RAS^G12P^, whereas they relatively more fluctuate in the transforming mutant. (Compare red and green in Fig. 1 and see Table S1). It is also interesting that G60 and Q61 residues are more mobile in wild-type protein than in H-RAS^G12P^ mutant despite the fact that both systems do not cause transformation in the cell (See Table S1). Moreover, residues 62–69, which are known to mediate binding of RAS to nucleotide exchange factor, namely GEF^37^, also relatively less fluctuate in the non-transforming mutant compared to wild-type RAS (See Table S2), which might explain why H-RAS^G12P^ has lower dissociation constant for GDP^16^. Consequently, tight binding between GEF and RAS might trigger rapid nucleotide exchange in the non-transforming mutant than wild type protein. In addition, these residues fluctuate more in GDP-bound H-RAS^WT^ than in GDP-bound H-RAS^G12P^ mutant suggesting that the nucleotide binding pocket might be intrinsically more stable in non-transforming mutant independent of the phosphorylation status of the nucleotide (See Fig. S1). Besides mutants, we also compared fluctuation patterns of H-RAS^WT^ and GAP-bound H-RAS^WT^ and showed that the presence of the effector further stabilizes T35, G60 and Q61 residues. Interestingly, however, Y32 residue highly fluctuates in H-RAS^G12P^ mutant and in GAP-bound RAS than the other systems.

### The catalytically important residues are positioned closer to the nucleotide in the non-transforming mutant independent of the phosphorylation state of the nucleotide

In a recent ^31P^NMR study^38,39^, GppNHp-bound H-RAS^WT^ has shown to be found in a mixture of different conformational states, namely State1 (inactive state) and State2 (active state) in solution. The active state is described by complete interaction between T35, G60, and *γ*-phosphate of GTP. The inactive state is further grouped into three sub-states, namely, inactive state 1, 2 and 3. In the first one, interaction between nucleotide, T35 and G60 is completely lost, whereas in the second one, interaction between G60 and nucleotide is maintained but the one formed between T35 and the nucleotide is lost. The inactive state 3 is discovered in a computational study which was conducted by Nussinov *et al*., where interaction between *γ*-phosphate of GTP and T35 is maintained, but the one formed between G60 and the nucleotide is lost^8,40^.

**Figure 2:**
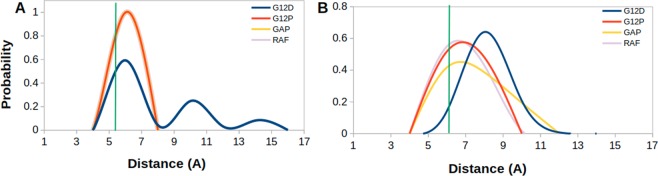
Probability distributions of two atom-pairs distances in GTP-bound states. (**A**) Distance between side chain oxygen atom of T35 and P*β* atom of GTP, namely Distance-1 and (**B**) Distance between backbone amide of G60 and P*β* atom of GTP, namely Distance 2, was measured using MD trajectories of corresponding systems. Green lines correspond to distances which are measured using crystal structure of GppNHp-bound H-RAS^WT^ (PDB ID:5P21).

**Figure 3:**
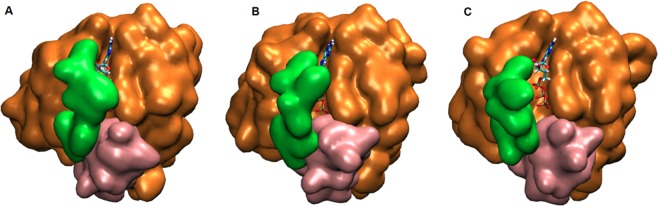
Representative structures that correspond to different states of the nucleotide binding pocket were taken from trajectories of GTP-bound systems. (**A**) State 2 (closed nucleotide binding pocket), (**B**) Inactive state 3, and (**C**) Inactive state 1 (exposed nucleotide binding pocket). Protein is shown in surface whereas GTP is shown in licorice representation. Switch I is shown in green, Switch II is shown in pink and rest of the protein is shown in orange color.

**Figure 4:**
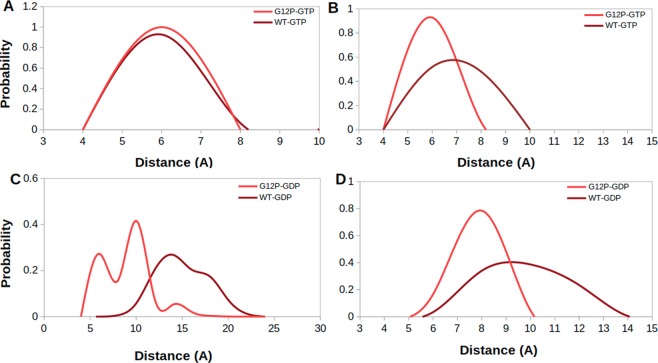
Probability distributions of two atom-pairs distances which were measured between (**A**,**C**) side chain oxygen atom of T35 and P*β* atom of the nucleotide (**B**,**D**) backbone amide of G60 and P*β* atom of the nucleotide.

In this part, we investigated conformational state of the nucleotide binding pocket of the systems using the same atom pair (T35-G60) given above. To do so, we calculated probability distributions of two atom-pairs distances between side chain oxygen of T35 and GTP P*β* atom; namely Distance 1, and between backbone amide of G60 and GTP P*β* atom; namely Distance 2. For comparison, we utilized corresponding distances in crystal structures of GppNHp-bound H-RAS^WT^ (PDB ID:5P21)^41^ complex, where Distance 1 and Distance 2 were measured as 5.5 Å and 6.2 Å, respectively. In general, the nucleotide is tightly coordinated by T35 and G60 residues in all of the systems, except H-RAS^G12D^, as evident from sampling relatively longer distance values in Distance 1 and 2 (See Fig. 2). Similar coordination is also observed for K-RAS4B^G12D^ mutant^8,40^. The transforming mutant resembles inactive State 1 with an open nucleotide binding pocket (longer Distance 1 & 2 values) (See Fig. 3C), whereas non-transforming mutant resembles State2 (shorter Distance 1 & 2 values) (See Fig. 3A), which corresponds to the active state with a compact nucleotide binding pocket. In addition, RAF-RBD-bound system resembles State 2, whereas GAP-bound system resembles a mixture of State 2 and Inactive State 3 (shorter Distance 1 and longer Distance 2 values), albeit with low probability, where interaction between P*β* atom of the nucleotide and backbone amide of G60 is lost but the one formed between T35 and the nucleotide is maintained (See Fig. 3B). We also investigated the impact of phosphorylation status of the nucleotide on the conformational state of the catalytic site in non-transforming mutant and H-RAS^WT^. To do so, we compared probability distributions for the same atom pairs as given in Fig. 2. We showed that exchange of GDP by GTP causes tight coordination of the nucleotide by both T35 and G60 which is evident by left shifts in Distance 1 & 2 values in both H-RAS^G12P^ mutant and H-RAS^WT^ (Compare A–C and B–D in Fig. 4), which is similar to what is observed for K-RAS4B^G12D^ and K-RAS4B^G12C^ mutants^40^. Moreover, T35 and G60 sample shorter distances from the nucleotide in both GDP- and GTP-bound H-RAS^G12P^ mutant compared to H-RAS^WT^ which might expedite the organization of the catalytically important residues around the nucleotide binding pocket.

### Dynamics of Q61 gives an insight into transforming capacity of systems

**Figure 5:**
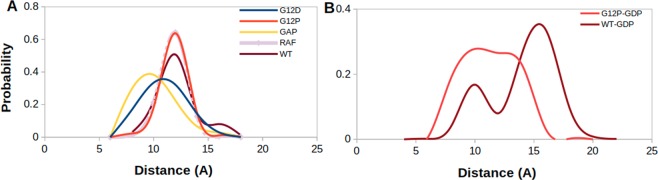
Probability distributions of two atom-pairs distances which were measured between side chain oxygen atom of Q61 and P*β* atom of the nucleotide in (**A**). GTP-bound H-RAS^G12D^, H-RAS^G12P^, GAP-bound H-RAS^WT^, RAF-RBD-bound H-RAS^WT^, and H-RAS^WT^ (**B**). GDP-bound H-RAS^G12P^ and H-RAS^WT^ proteins.

To have more insight on the catalytic activity of systems, we investigated orientational dynamics of Q61, which is known to contribute to GTPase activity of RAS. To do so, we computed probability distributions of atom-pair distances which are measured between the side chain oxygen atom of Q61 and P*β* atom of the nucleotide as shown in Fig. 5.

We showed that Q61 is positioned relatively closer to the nucleotide in GAP-bound H-RAS^WT^, (See yellow in Fig. 5), which is expected since it represents the catalytically-active state of RAS. On the other hand, the same residue is located farther from the nucleotide binding pocket in signaling-active RAF-RBD-bound system which prevents GTP hydrolysis (See purple in Fig. 5). Q61 samples longer distances from the nucleotide in the transforming mutant (See blue in Fig. 5) than GAP-bound system, which explains why it cannot catalyze hydrolysis and causes transformation in the cell. It samples even longer distances from the nucleotide in H-RAS^G12P^ than H-RAS^G12D^, which is similar to RAF-RBD-bound H-RAS^WT^ (See red in Fig. 5). This is an interesting observation because the catalytically important residue is positioned closer to the nucleotide in the transforming mutant than the non-transforming mutant. We also investigated the impact of the phosphorylation status of the nucleotide on dynamics of Q61 by measuring the same distance in both GDP- and GTP-bound protein. We showed that exchange of GDP by GTP increases the coordination of the nucleotide in both H-RAS^WT^ and H-RAS^G12P^ as evident from relatively shorter distance values measured for GTP-bound state (compare Fig. 5A and B). Lastly, it is also important to emphasize that Q61 samples shorter distance values in GDP-bound H-RAS^G12P^ compared to GDP-bound H-RAS^WT^ (Fig. 5B) as also seen for T35 and G60 residue.

### Dynamics of Y32 provides mechanistic insight into molecular switch function of H-RAS^G12P^ mutant

**Figure 6:**
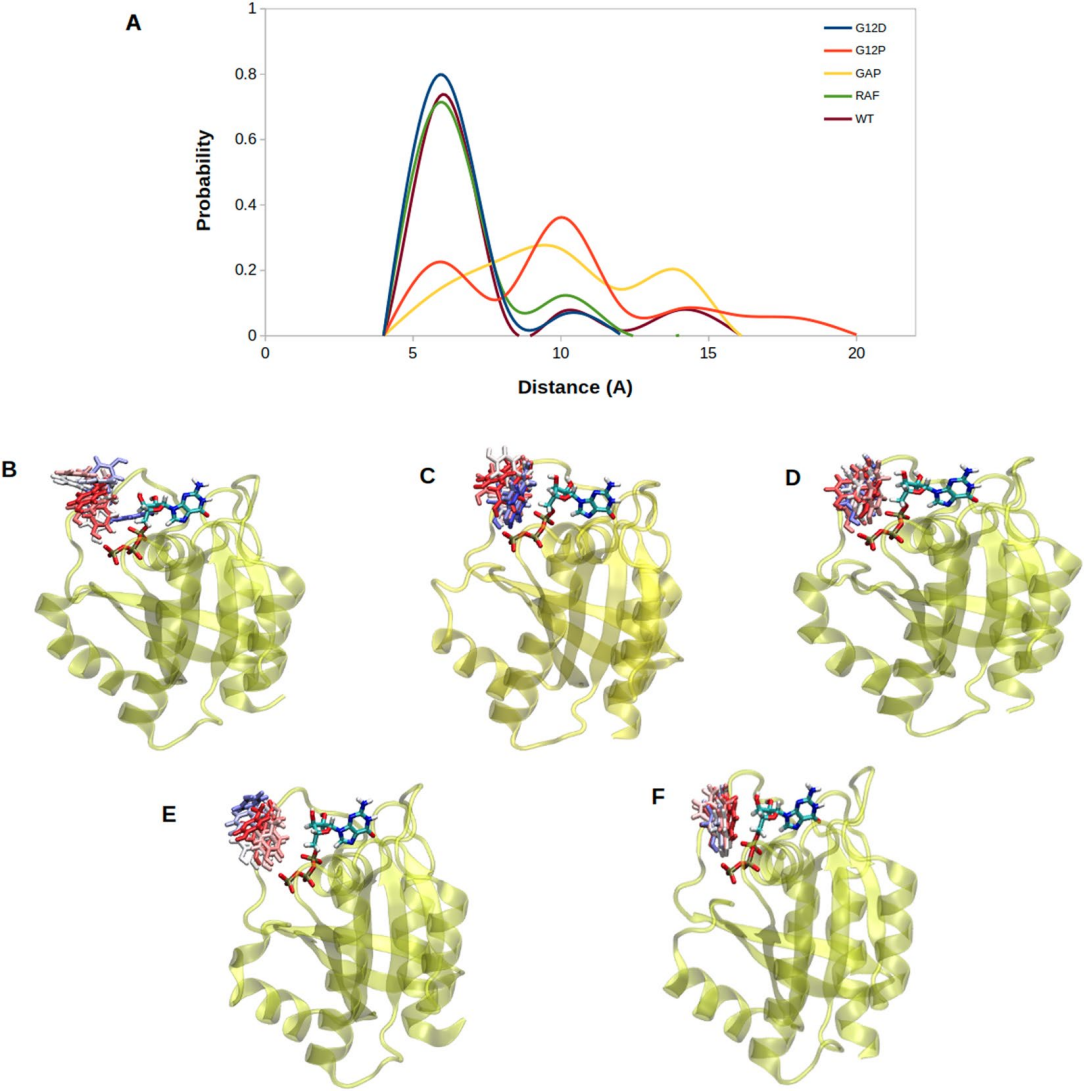
(**A**) Probability plots of distance distributions were measured between side chain oxygen atom of Y32 and P*β* atom of GTP by using MD trajectories of the systems. Snapshots show the orientation of Y32 with respect to nucleotide binding pocket in (**B**) H-RAS^G12P^, (**C**) H-RAS^WT^, (**D**) H-RAS^G12D^, (**E**) GAP- bound H-RAS^WT^, (**F**) RAF-RBD-bound H-RAS^WT^. Y32 is colored with respect to the frame number in corresponding MD trajectories. Y32 and GTP are shown in licorice, whereas protein is shown in yellow and new cartoon representation.

As comparative analysis of RMSF plots reveals (See Fig. 1), Y32 highly fluctuates in H-RAS^G12P^ mutant as well as in GAP-bound H-RAS^WT^, whereas it is quite stable in the transforming mutant, RAF-RBD-bound H-RAS^WT^ and H-RAS^WT^. Detailed analysis of the trajectories showed that Y32 forms hydrogen bond with *γ*-oxygen of GTP in the transforming mutant and RAF-RBD-bound H-RAS^WT^, so it is positioned closer to the nucleotide throughout the trajectory as shown in Fig. 6 (See A (blue and green, respectively), D and F). Here, the electrostatic repulsion between negatively charged aspartic acid residue and GTP in H-RAS^G12D^ mutant causes this residue to be repelled from the nucleotide binding site, thus leaving a space for Y32 to interact with GTP. On the other hand, the abovementioned hydrogen bond is not formed in the non-transforming mutant and GAP-bound H-RAS^WT^ as it is evident from wider distance distributions which are measured between the side chain of Y32 and P*β* atom of GTP (See Fig. 6 (See A (red and yellow), B and E). Detailed analysis of the trajectory of non-transforming mutant showed that the proline residue at 12^th^ position in H-RAS^G12P^ mutant is closely positioned to GTP, thus leaving partly no space for Y32 to interact with the nucleotide.

**Figure 7:**
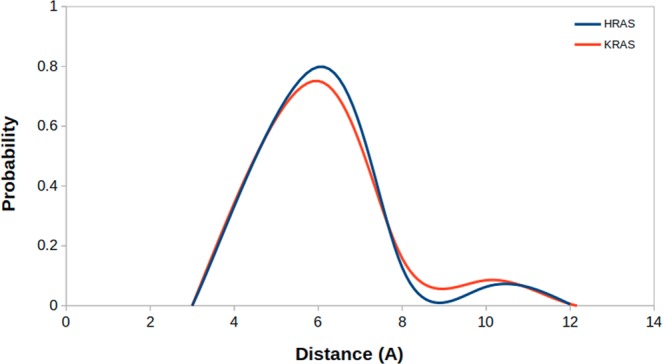
Probability plot shows distance distributions which were measured between side chain oxygen of Y32 and P*β* atom of GTP in H-RAS^G12D^ and K-RAS4B^G12D^ mutants.

Considering the fact that RAF-RBD-bound H-RAS^WT^ represents the signaling-active state of RAS, it is expected to observe that Y32 makes close interaction with the nucleotide, thus preventing access to GTP for hydrolysis. Similarly, Y32 is not accessible also in the transforming mutant, making close interaction with the GTP, thus preventing access to the nucleotide and locking RAS in the “on” state. On the other hand, Y32 is dominantly exposed in the catalytically-active GAP-bound H-RAS^WT^, which is also expected because, in this way, GTP can be accessed by the nucleophilic water, thus triggering hydrolysis of the nucleotide. Interestingly, in the non-transforming H-RAS, Y32 samples both states (“*exposed*” and “*non-exposed*”), albeit with low probability of the “*non-exposed*” state. However, Y32 is exposed in H-RAS^WT^ upon GAP-binding as it is found in the “*exposed*” state in GAP-bound H-RAS^WT^ but not in H-RAS^WT^ trajectories. Moreover, we also showed that dynamics of Y32 in H-RAS^G12P^ mutant is modulated by the phosphorylation status of the nucleotide as Y32 can be closely positioned to the nucleotide in GDP-bound RAS (See Fig. SI-2). Lastly, it is also important to emphasize that Y32 is positioned closer to the nucleotide in another mutant RAS subtype as well, namely, K-RAS4B^G12D^ (PDB ID:4DSN)^18^ as shown in Fig. 7 suggesting that the incomplete opening of Y32 is conserved among different mutant RAS subtypes.

### Local dynamic properties also emerge in dominant global motions of systems

**Figure 8:**
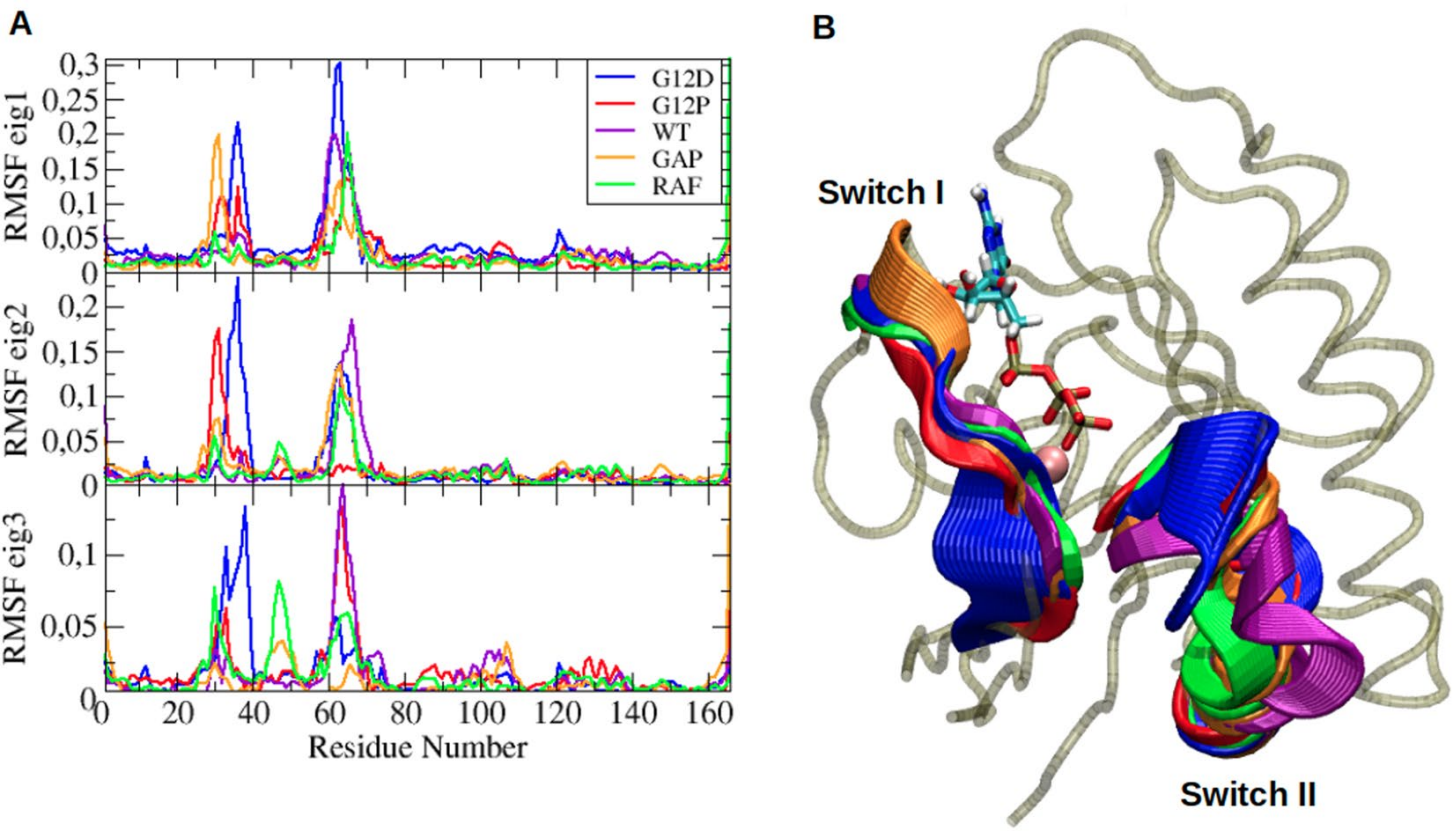
(**A**) Comparison profile of fluctuations along first three essential eigenvectors. (**B**) Extreme structures obtained along eigenvector 1 were shown for H-RAS^G12D^ (blue), H-RAS^G12P^ (red), H-RAS^WT^ (purple), GAP-bound H-RAS^WT^ (orange), and RAF-RBD-bound H-RAS^WT^ (green), where thickness of ribbons corresponds to the magnitude of contributions of residues to overall dynamics. Mg^2+^ ion is shown in pink and van der Waals while GTP is shown in licorice representation.

Beside exploring local structural/dynamics properties of the systems, we also investigated dominant collective motions in trajectories by means of essential dynamics analysis. Here, we depicted first three eigenvectors which cumulatively constitute 50% of the overall motion and highlight distinctions in dynamics of the systems as shown in Fig. 8. In general, we observed that local system-specific properties also emerge as dominant global motions in the systems, which are evident from fluctuation profiles obtained from projection of the trajectories along their first three essential eigenvectors (See Fig. 8A). According to that, Switch I and II regions dominate overall motion in transforming mutant as evidenced by higher fluctuation in these regions with respect to the nucleotide binding pocket. This is represented by extreme structures that are obtained by projection of trajectories along their first eigenvectors (See Fig. 8B). On the other hand, motion of Y32 contributes more to the overall motion in non-transforming mutant and GAP-bound H-RAS^WT^ (See red and orange in Fig. 8A).

## Discussion

To the best of our knowledge, this is the first study, where structural and dynamical properties of non-transforming H-RAS^G12P^ mutant have been revealed. Our results showed that glycine to proline mutation introduced stability to H-RAS^G12P^ as evident from lower flexibility of Switch I & II in this non-transforming mutant. Interestingly, despite the fact that both wild type protein and H-RAS^G12P^ do not cause transformation, the latter resembles more catalytically competent state than the former. In particular, relatively lower fluctuation of the catalytically important residue Q61 in both GDP- and GTP-bound states of H-RAS^G12P^ might expedite proper organization of the nucleotide binding pocket, thus increasing GTPase activity of non-transforming mutant compared to wild type protein, which agrees with experimental data^16^.

Apart from T35, G60 and Q61, we also observed remarkable differences in dynamics of Y32. Specifically, it is positioned closer to the nucleotide in signaling-active systems, which are represented by RAF-RBD-bound H-RAS^WT^ and H-RAS^G12D^, thus preventing accessibility of GTP for hydrolysis. On the other hand, it is located far from the nucleotide binding pocket in catalytically-active state, which is represented by GAP-bound H-RAS^WT^ complex. Interestingly, however; Y32 samples both of these orientations in H-RAS^G12P^. Considering the fact that orientational dynamics of Y32 is crucial for proper function of RAS^30,33^, understanding simultaneous sampling of “*exposed*” and “*non-exposed*” states of Y32 can provide insight into intrinsic molecular switch function of H-RAS^G12P^. Presumably, when Y32 is positioned closer to and Q61 is positioned far from the nucleotide H-RAS^G12P^ can conduct signal; however, when Y32 is exposed and Q61 is positioned closer to the nucleotide, non-transforming mutant catalyzes hydrolysis of GTP.

In light of these findings, we propose that Y32 can be used as an alternative site which can be targeted by small therapeutic molecules to displace it from the nucleotide binding pocket, thus introducing intrinsic GTPase activity to transforming RAS mutants that cannot bind to GAP. Moreover, we believe that mimicking structural and dynamical properties of H-RAS^G12P^ will improve success rates in drug discovery studies since it resembles catalytically competent state more than H-RAS^WT^.

## Methods

### System setup for MD simulations

Simulations of systems were performed as (a) guanosine triphosphate (GTP-) bound H-RAS^G12P^ (PDB ID: 1JAH)^17^, H-RAS^G12D^ (PDB ID: 1AGP)^16^, H-RAS^WT^ (PDB ID: 5P21)^41^, GAP-bound H-RAS^WT^ (PDB ID: 1WQ1)^42^, and RAF-RBD-bound H-RAS^WT^ (PDB ID:4G0N)^43^ (b) guanosine diphospate (GDP-) bound H-RAS^G12P^ and H-RAS^WT^ (PDB ID: 4Q21)^44^. Corresponding crystal structures were retrieved from Protein Data Bank (PDB). In order to prepare GTP and GDP-bound H-RAS^G12P^ systems, phosphomethylphosphonic acid guanylate ester (GCP) was converted to GTP and GDP by substituting C_3_B with O and removing *γ*-phosphate, sequentially. For H-RAS^G12D^ and H-RAS^WT^, conversion of phosphoaminophosphonic acid-guanylate ester (GNP) to GTP was done by interchanging N_3_B with O. As a side note, GDP bound H-RAS^WT^ does not require any manipulation since it has been resolved with GDP. For GAP-bound H-RAS^WT^, aluminum fluoride (AlF_3_) was extracted and GDP was exchanged with GTP. For RAF-bound H-RAS^WT^ system, acetate ion, (2R,3S) -1,4-dimercaptobutane-2.3-diol, and calcium ion were removed from the system, and GNP was substituted with GTP as it has done for both H-RAS^G12D^ and H-RAS^WT^. Lastly, GTP-bound K-RAS4B^G12D^ were prepared by removing 1,2-ethanediol and substituting GCP with GTP in the same way mentioned for H-RAS^G12P^. Also, crystal waters which are located within 5 Å of nucleotide were kept for all the systems. After these manipulations, protonation states of amino acids for all systems were assigned by using PropKa server^45^ at pH 7.4. By taking periodic boundary conditions (PBCs) into account, thickness of water layer was adjusted as 9 Å, except GAP- and RAF-RBD-bound H-RAS^WT^. For these systems, it was set as 11 Å and 13 Å, respectively. Furthermore, TIP3P^46^ water model was utilized, and systems were neutralized with NaCl.

### Simulation protocols

MD simulations were carried out by using Compute Unified Device Architecture (CUDA) version of Nano Scale Molecular Dynamics (NAMD) with the help of graphical processing units (GPUs) acceleration where CHARMM 36 was used to model proteins, ligands, and ions^47,48^. Systems were minimized for 4.8 picoseconds (ps) both in NVT and NPT ensembles. Temperature and pressure were set 310 K and 1 atm, respectively. Time step was adjusted to 2 femtoseconds (fs) in order to capture the fastest motions within systems. Cut off value for non-bonded interactions was set to 12 Å and long-range electrostatic interactions were computed by using particle mesh Ewald (PME). Furthermore, trajectory snapshots were saved in every 5 ps. Both GDP/GTP-bound H-RAS^WT^ and H-RAS^G12P^ were simulated for total of 4.8 *μ*s, whereas GTP-bound H-RAS^G12D^, RAF-RBD-bound and GAP-bound H-RAS^WT^ were performed in total 3.6 *μ*s. Lastly, MD simulation of GTP-bound K-RAS4B^G12D^ was performed for 0.6 *μ*s. Here, we performed two separate simulations for the systems, each of which was started with different initial velocity. The results were presented as a combination of these two replicas.

### Root-mean-square fluctuation (RMSF)

Root-mean-square fluctuation was computed as following:

$${\rm{RMSF}}=\sqrt{(1/N)\,\sum _{n=1}^{N}\,({X}_{{\rm{i}}}(n)-\overline{{X}_{{\rm{i}}}})}$$

where N was duration of simulation, X_i_(n) coordinates of backbone atom X_i_ at time n. Sum of squared difference of mean coordinate X_i_ and X_i_(n) was computed and divided by the duration of simulation. Lastly, square root of the result was taken. For this analysis, ‘gmx rmsf’ module of GROMACS was utilized to explore dynamic profiles by assessments of fluctuations of residues^49^.

### Principal component analysis (PCA)

Apart from exploring local dynamic and structural properties of the systems, dominant collective motions occurring in the trajectories were demystified by means of PCA. For this, trajectories were aligned with respect to the C*α* atoms of reference structure.

$${{\rm{C}}}_{{\rm{ij}}}=\langle {{\rm{M}}}_{{\rm{ij}}}{{\rm{\Delta }}{\rm{r}}}_{{\rm{i}}}{{\rm{\Delta }}{\rm{r}}}_{{\rm{j}}}\rangle $$

where C_ij_ corresponds to covariance matrix. A change in position from time-averaged structure for each coordinates of all atoms i and j was denoted as M_ij_Δr_i_Δr_j_.

Covariance matrices were generated as shown in above.

$${\rm{Cv}}={\delta }^{2}v$$

Diagonalisation of covariance matrices provided a set of eigenvalues and eigenvectors *δ*^2^, and v, respectively.

Computation and diagonalisation of covariance matrices were done by making use of ‘gmx covar’ module of GROMACS while ‘gmx anaeig’ module of GROMACS was used to obtain eigenvectors and eigenvalues from diagonalized covariance matrices^49^.

## Supplementary information

Catalytically Competent Non-transforming HRAS<sup>G12P</sup> Mutant Provides Insight into Molecular Switch Function and GAP-independent GTPase Activity of RAS
