# Supplementary material for: Catalytically Competent Non-transforming H-RASG12P Mutant Provides Insight into Molecular Switch Function and GAP-independent GTPase Activity of RAS
Source: Sci Rep. 2019 Jul 29;9:10967. doi: 10.1038/s41598-019-47481-1 (PMC6662853; doi:10.1038/s41598-019-47481-1)
Supplement: Supplementary file 1 — Catalytically Competent Non-transforming HRASG12P Mutant Provides Insight into Molecular Switch Function and GAP-independent GTPase Activity of RAS [file 41598_2019_47481_MOESM1_ESM.pdf]

Supporting Information

Catalytically Competent Non-transforming HRAS^{G12P} Mutant Provides Insight into Molecular Switch Function and GAP-independent GTPase Activity of RAS

Metehan Ilter[1] and Ozge Sensoy[2,*]

[1]Istanbul Medipol University, The School of Engineering and Natural Sciences, Department of Biomedical Engineering, Istanbul, 34810, Turkey

[2]Istanbul Medipol University, The School of Engineering and Natural Sciences, Department of Computer Engineering, Istanbul, 34810, Turkey

Corresponding Author: *O.S.: e-mail, osensoy@medipol.edu.tr

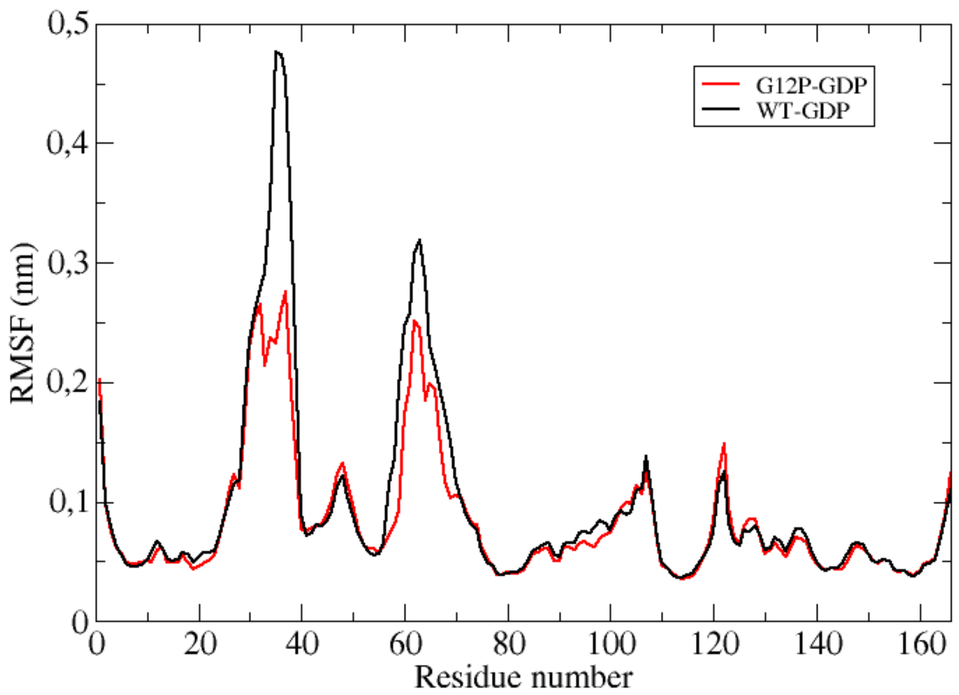

Figure S1. Root-mean-square fluctuations of GDP-bound HRAS^{G12P} (PDB ID: 1JAH) and GDP-bound HRAS^{WT} (PDB ID: 4Q21) computed from molecular dynamics trajectories.

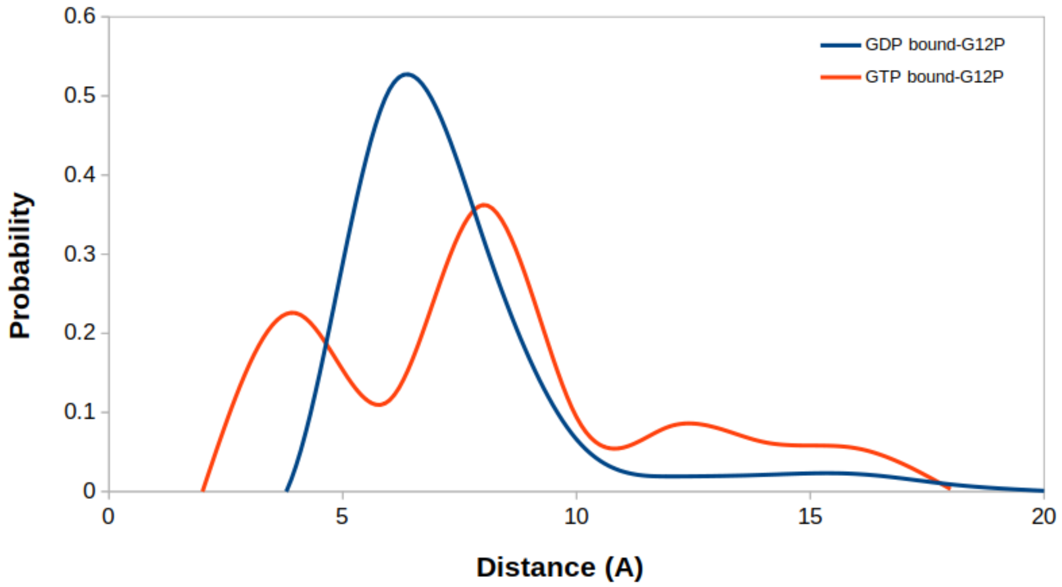

Figure S2. Probability plots of distance distributions which were measured between side chain oxygen atom of Y32 and Pβ atom of GTP/GDP calculated from MD trajectories of non-transforming mutant.

Table S1. RMSF values of residues that are known to be responsible for function of the protein.

Residue / RMSF (nm)	HRAS^{G12D}	HRAS^{G12P}	HRAS^{WT}	GAP-bound HRAS^{WT}	RAF-RBD-bound HRAS^{WT}
**Y32**	0.1398	0.2028	0.1038	0.1516	0.1101
**T35**	0.2791	0.1002	0.0882	0.0551	0.0635
**G60**	0.1844	0.0793	0.1949	0.1471	0.0917
**Q61**	0.2407	0.1026	0.2247	0.1538	0.1078

Table S2. RMSF values of residues that mediate interaction of RAS with GEF.

System/RMSF (nm)	62	63	64	65	66	67	68	69
**WT**	0.247	0.261	0.2637	0.2689	0.2667	0.2159	0.1613	0.123
**G12P**	0.1505	0.1829	0.2113	0.191	0.1846	0.1381	0.111	0.0978
